# Strong influence of livestock environments on the emergence and dissemination of distinct multidrug-resistant phenotypes among the population of non-typhoidal *Salmonella*

**DOI:** 10.1371/journal.pone.0179005

**Published:** 2017-06-07

**Authors:** Ran An, Sahar Alshalchi, Peter Breimhurst, Jeannette Munoz-Aguayo, Christian Flores-Figueroa, Sinisa Vidovic

**Affiliations:** 1 Department of Veterinary and Biomedical Sciences, University of Minnesota, Saint Paul, United States of America; 2 College of Biological Sciences, University of Minnesota, Minneapolis, United States of America; 3 Mid-Central Research and Outreach Center, University of Minnesota, Willmar, United States of America; Purdue University, UNITED STATES

## Abstract

The problem of emergence and dissemination of multidrug resistance, especially among Gram-negative bacteria, has reached alarming levels. This increases the need to develop surveillance methods that more effectively and accurately provide information about the emergence and spread of multidrug-resistant organisms. In this study, using a well-defined population of non-typhoidal *Salmonella* (NTS) isolates associated with avian, bovine and porcine hosts, we found that the livestock environments had a specific (P < 0.005) and profound (P < 0.005) effect on the evolution of multidrug-resistant phenotypes among population of NTS isolates. The MDR pattern containing penicillins, tetracyclines and macrolides and the evolving counterparts (i.e., penicillins, tetracyclines and macrolides + other antibiotic classes) were significantly (P < 0.005) associated with NTS isolates of porcine origin. Similarly, MDR patterns containing folate pathway inhibitors, macrolides and aminocyclitol or containing penicillins, cephalosporins, tetracyclines, phenicols and macrolides were significantly associated with avian (P < 0.005) and bovine (P < 0.005) NTS isolates, respectively. Furthermore, STRUCTURE, an evolutionary analysis, clearly showed that the host origin (i.e., livestock environment), and not the genetic background of different NTS serovars, was the most determinative factor for acquisition and spread of MDR phenotypes. In addition, we described a novel non-synonymous mutation, located outside of the QRDR at position 864 of GyrA, that was likely associated with fluoroquinolone resistance.

## Introduction

Non-typhoidal *Salmonella* (NTS) remains a major food-borne pathogen worldwide [1]. Infections with this group of *Salmonella* have not decreased over the past 15 years in the United States [2, 3]. The global situation is even worse. Over 1.3 billion humans experience salmonellosis (i.e., infection caused by NTS) annually, with approximately three million deaths throughout the world [4, 5]. Recently, it has been reported that NTS is strongly associated with a life threatening, extra-intestinal invasive disease, leading to bacteremia and systematic infections, especially among immunocompromised individuals [6] (hereafter referred to as invasive NTS [iNTS]). Most iNTS strains are associated with multidrug resistance [6], which subsequently increases rates of hospitalizations, blood stream infections and mortality rates [7]. Association between multidrug resistance (MDR) (i.e., resistance to at least one agent in three antimicrobial categories [8]) and iNTS has been considered a major public health concern [9]. It has been estimated that iNTS annually causes approximately 680,000 deaths worldwide [10]. Recently, in an analysis of whole-genome sequences of 675 isolates of *S*. *enterica* serovar Enteritidis from 45 countries, Feasey et al. [6] revealed the existence of a global epidemic lineage and two new lineages that were geographically restricted to certain regions of Africa. Both African epidemic lineages are associated with an expended multidrug-resistance-augmented virulence plasmid and a novel prophage repertoire, similar to other lineages of NTS associated with invasive disease in Africa [11, 12]. Another group of authors, while examining a global collection of typhoidal *Salmonella* isolates (i.e., *Salmonella enterica* serovar Typhi), identified a single dominant MDR lineage, H58, that has emerged and spread throughout Asia and Africa over the last 30 years [13]. This is particularly concerning because H58 lineages are displacing antibiotic-sensitive isolates, further limiting treatment options for invasive *Salmonella* disease [13].

To treat invasive *Salmonella* diseases such as bacteremia and meningitis, fluoroquinolones are used as the first-line antibiotics. Because of the side effects, fluoroquinolones are not prescribed for the treatment of invasive *Salmonella* diseases in children; third-generation cephalosporins are of particular clinical importance to treat this invasive disease in children [7]. Soon after the introduction of fluoroquinolones, resistance and decreased susceptibility to this drug among populations of NTS have been reported in the USA [14], Southeast Asia [15, 16] and Denmark [17]. It is believed that emergence and dissemination of antimicrobial-resistant phenotypes occur frequently in zoonotic bacteria, such as NTS, due to the use of antibiotics for growth promotion, chemotherapy and prophylaxis in livestock [18, 19]. However, relatively little is known about the emergence and evolution of MDR phenotypes in NTS strains associated with different food-producing animals (i.e., livestock environments). Here, we have used phylogenetic and population structure analysis in parallel with antimicrobial susceptibility testing and sequencing the full-length *gyrA* and *gyrB* genes of a large collection of NTS isolates obtained from avian, bovine and porcine hosts. This study aimed to reveal the emergence and evolution of multidrug and fluoroquinolone resistance among different host-associated NTS populations. Our ultimate goal was to investigate the effect of different livestock environments (i.e., avian, porcine and bovine) on emergence and evolution of MDR in non-typhoidal *Salmonella*.

## Results

### Antimicrobial susceptibility of the NTS isolates

All isolates (n = 240) were resistant to at least one antibiotic. The phenotype most commonly distributed among the collection of NTS isolates was resistance to macrolides (100%; n = 240) (Table 1). All other antimicrobial categories demonstrated significant association with certain host-origin based groups. Chi-squared test revealed a significant (P < 0.005) association of the tetracycline resistance phenotype with the porcine (100%; n = 80) and bovine (63.7%; n = 51) isolates compared to that of the avian (11.2%; n = 9) isolates. The resistance to cephalosporin (i.e., third generation) was significantly (P < 0.005) associated with the bovine isolates (52.5%; n = 42), compared to the porcine (10%; n = 8) and the avian (15%; n = 12) isolates. Another highly significant (P < 0.005) antimicrobial resistance phenotype association was observed for the folate pathway inhibitors antimicrobial category. Most NTS strains (77.5%; n = 62) of avian origin showed resistance to folate pathway inhibitors (e.g., trimethoprim / sulfamethoxazo), whereas fewer bovine (16.2%; n = 13) and porcine (25%; n = 20) isolates shared the same phenotype (i.e., resistance to folate pathway inhibitors).

**Table 1:** Antibiogram of the non-typhoidal *Salmonella* (NTS) isolates obtained from different host origin.

Antimicrobial groups	Non-typhoidal *Salmonella* host origin									
	Avian			Bovine			Porcine			
	Resistant	Intermediate	Susceptible	Resistant	Intermediate	Susceptible	Resistant	Intermediate	Susceptible	*P* value
**Penicillins**	22 (27.5%)		58 (72.5%)	45 (56.2%)		35 (43.7%)	66 (82.5%)		14 (17.5%)	2.2 e-11
**Cephalosporin** (third generation)	12 (15%)	4 (5%)	64 (80%)	42 (52.5%)	2 (2.5%)	36 (45%)	8 (10%)	1 (1.25%)	71 (88.7%)	1.6 e-10
**Tetracyclines**	9 (11.2%)		71 (88.7%)	51 (63.7%)		29 (36.2%)	80 (100%)			1.9 e-28
**Fluroquinolones**			80 (100%)		8 (10%)	72 (90%)	11 (13.7%)	11 (13.7%)	58 (72.5%)	9.8 e-06
**Phenicols**	4 (5%)	3 (3.7%)	73 (91.2%)	47 (58.7%)	9 (11.2%)	24 (30%)	28 (35%)	4 (5%)	48 (60%)	3.8 e-12
**Folate pathway inhibitors** (trimethoprim / sulfamethoxazo)	62 (77.5%)		18 (22.5%)	13 (16.2%)		67 (83.7%)	20 (25%)		60 (75%)	1.1 e-16
**Macrolides**	80 (100%)			80 (100%)			80 (100%)			1.00
**Aminocyclitol**	37 (46.2%)	6 (7.5%)	37 (46.2%)		22 (27.5%)	58 (72.5%)	37 (46.2%)	19 (23.7%)	24 (30%)	2.4 e-12
**Total**	35.3%	2%	62.7%	43.5%	6.4%	50.1%	51.5%	5.5%	43%	

In total, 32 MDR patterns were observed among the collection of 240 NTS isolates. Out of 32 MDR patterns, 21 were observed among the porcine isolates followed by thirteen and seven MDR patterns among the avian and bovine isolates, respectively (Table 2). Eight MDR patterns were shared among the isolates of porcine, bovine and avian origin, whereas sixteen MDR patterns were unique for the porcine isolates, followed by seven and one MDR patterns unique for the avian and bovine isolates, respectively (Table 2).

**Table 2:** Distribution of MDR within the collection of NTS isolates obtained from avian, bovine and porcine hosts.

Multidrug resistance pattern	No. (%) of isolates positive for MDR			
	Avian (*n* = 51)	Bovine (*n* = 48)	Porcine (*n* = 78)	*P* value
Penicillins, tetracycline, macrolide, cephalosporin	2 (3.9)	3 (6.2)	2 (2.6)	0.86
**Penicillins, tetracycline, macrolide**^c^			26 (33.3)	0.00000
Penicillins tetracycline, macrolide, phenicol, FP inhibitors^a^, aminocyclitol			5 (6.4)	0.0067
Penicillins, tetracycline, macrolide, FP inhibitors, aminocyclitol			5 (6.4)	0.0067
Penicillins tetracycline, macrolide, phenicol, aminocyclitol			8 (10.2)	0.00033
Penicillins, tetracycline, macrolide, phenicol		2 (4.2)	6 (7.7)	0.030
Penicillins, tetracycline, macrolide, phenicol, FP inhibitors		1 (2)	1 (1.3)	NA
Tetracycline, macrolide, aminocyclitol, phenicol, FP inhibitors	1 (1.9)		2 (2.6)	NA
Tetracycline, macrolide, aminocyclitol, phenicol			1 (1.3)	NA
Penicillins tetracycline, macrolide, FP inhibitors			1 (1.3)	NA
Penicillins tetracycline, macrolide, aminocyclitol			2 (2.6)	NA
Tetracycline, macrolide, aminocyclitol			7 (9)	0.00091
Penicillins, tetracycline, macrolide, cephalosporin, FP inhibitors, aminocyclitol	1 (1.9)		1 (1.3)	NA
Penicillins, tetracycline, macrolide, cephalosporin, FP inhibitors, aminocyclitol, phenicol			2 (2.6)	NA
Penicillins, tetracycline, macrolide, cephalosporin, FQ^b^, phenicol, aminocyclitol			1 (1.3)	NA
Penicillins, tetracycline, macrolide, cephalosporin, FP inhibitors, phenicol, aminocyclitol			1 (1.3)	NA
Penicillins, tetracycline, macrolide, phenicol, FQ			1 (1.3)	NA
Penicillins, tetracycline, macrolide, FQ, phenicol, aminocyclitol			1 (1.3)	NA
Tetracycline, macrolide, FQ			2 (2.6)	NA
Penicillins, tetracycline, macrolide, FQ			1 (1.3)	NA
Penicillins, tetracycline, macrolide, FQ, FP inhibitors			2 (2.6)	NA
**Penicillins, cephalosporin, tetracycline, phenicol, macrolide**^d^	1 (1.9)	26 (54.1)		0.00000
Penicillins, cephalosporin, tetracycline, phenicol, macrolide, FP inhibitors	1 (1.9)	11 (22.9)		0.000096
Tetracycline, macrolide, phenicol	1 (1.9)	4 (8.3)		0.074
Tetracycline, macrolide, phenicol, FP inhibitors		1 (2)		NA
**FP inhibitors, macrolides, aminocyclitol**^e^	27 (52.9)			0.00000
FP inhibitors, macrolides, aminocyclitol, penicillins, cephalosporin	1 (1.9)			NA
FP inhibitors, macrolides, aminocyclitol, penicillins	5 (9.8)			0.0067
FP inhibitors, macrolides, penicillins, cephalosporin	4 (7.8)			0.018
,Penicillins, cephalosporin, macrolide	2 (3.9)			NA
FP inhibitors, macrolides, aminocyclitol, tetracycline	2 (3.9)			NA
FP inhibitors, macrolides, penicillins	3 (5.9)			0.049

^a^ Folate pathway inhibitors (trimethoprim / sulfamethoxazo).

^b^ Fluoroquinolones.

^c^ The most common MDR pattern associated with the porcine strains.

^d^ The most common MDR pattern associated with the bovine strains.

^e^ The most common MDR pattern associated with the avian strains.

### Association of non-synonymous point mutations in the *gyrA* and *gyrB* with quinolone resistance in NTS

In total, 74 single nucleotide mutations were identified in the entire *gyrA* gene from 240 NTS isolates. Most of these point mutations were silent, and only five mutations resulted in amino acid substitutions in the GyrA protein. The most common amino acid substitution was observed at position 868, where serine (S) was substituted by asparagine (N) in 49 (20.4%) NTS isolates. This amino acid substitution occurred in most quinolone susceptible isolates, 40/49 (81.6%), and few, 9/49 (18.4%), quinolone resistant isolates, indicating that this mutation was not associated with quinolone resistance. Another amino acid substitution, not relevant to quinolone resistance, was found at position 759 of GyrA. This amino acid substitution, where an aspartic acid (D) was changed into a glutamic acid (E), specifically occurred among fifteen isolates of *Salmonella enterica* serovar Montevideo, strongly indicating that this amino acid substitution was related to the serovar. The remaining three amino acid substitutions were associated with quinolone resistance. Among this group, the most common amino acid substitution occurred in the quinolone resistance-determining region (QRDR) of *Salmonella enterica* spp. (i.e., amino acids 67–112) at position 87 of the GyrA. Two isolates, serovar Dublin (bovine isolate) with an intermediate phenotype and serovar Typhimurium var 5 (porcine isolate) resistant to quinolone had a substitution of D for tyrosine (Y) at position 87. Two more isolates, serovars Dublin (bovine isolates) and Typhimurium var 5- (porcine isolate) had a substitution of D for N at the same position. These two isolates showed decreased susceptibility to quinolone with the intermediate resistance phenotype. Another mutation in QRDR occurred at position 83. Five isolates from serovar Dublin (bovine isolates) with the quinolone intermediate phenotype had a substitution of S for phenylalanine (F) at position 83. Only one likely quinolone resistance-associated amino acid substitution outside of the QRDR occurred in an isolate, serovar Typhimurium var 5- (porcine isolate), at position 864 where P was substituted for S. For the remaining eight isolates of serovar Typhimurium var 5-, resistant to quinolone, no amino acid substitution was identified in the GyrA.

In 160 isolates (80 avian isolates and 80 porcine isolates), no single mutation was observed in the *gyrB*, whereas in 80 isolates of bovine origin, there were 73 single nucleotide mutations throughout the *gyrB* gene. These 73 point mutations contained only two non-silent mutations that separated the population of bovine isolates into three groups, specifically based on the corresponding serovars. No amino acid substitution in the GyrB was related to quinolone resistance.

### Phylogeny of the NTS population

**Fig 1:**
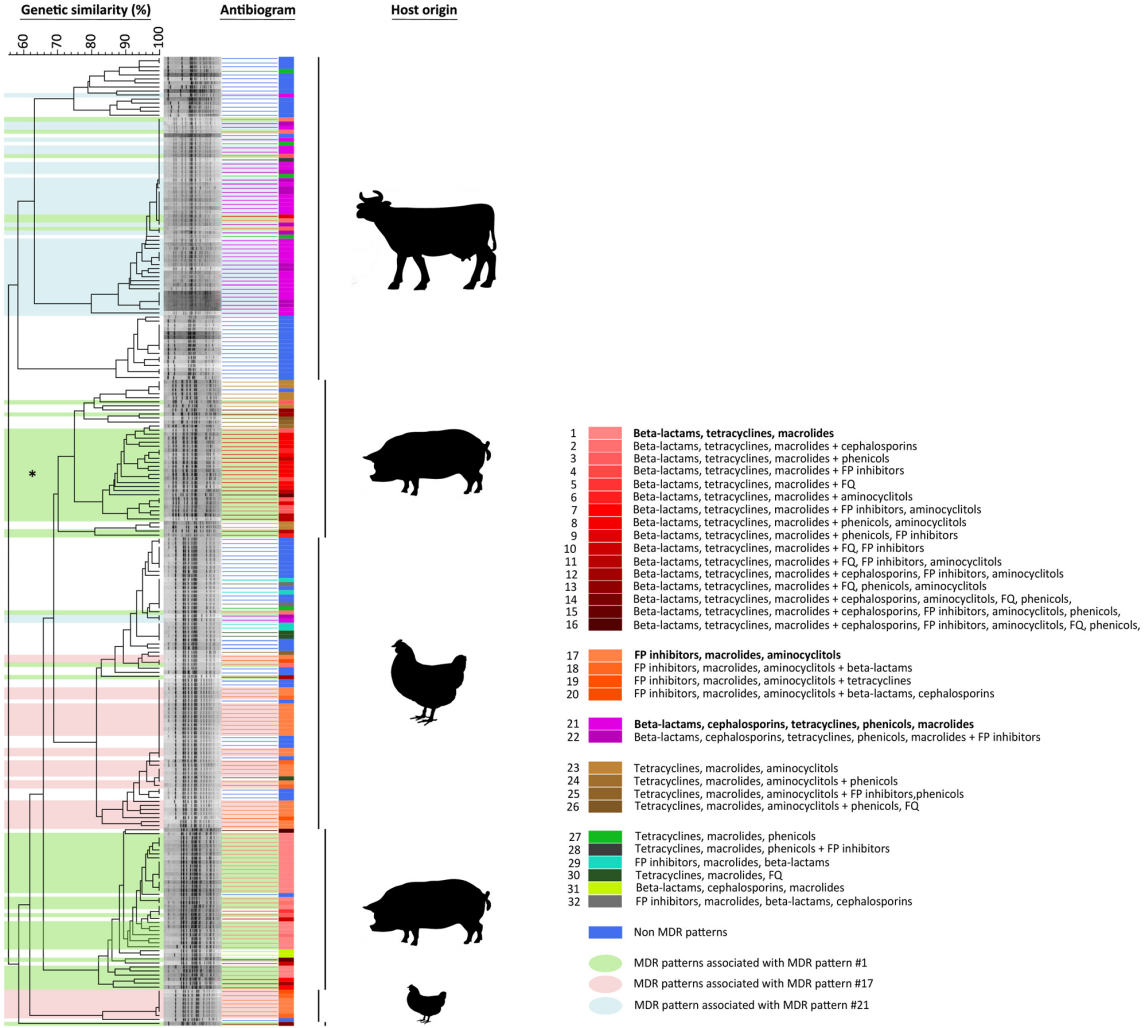
Dendrogram of 240 non-typhoidal *Salmonella* isolates depicting multidrug-resistant phenotype distribution across *S*. *enterica* pulsotypes. Each isolate was presented by the corresponding antibiogram. The shaded rectangles mark three major MDR patterns with the evolved counterparts as green (i.e., penicillins, tetracyclines, and macrolides with fifteen other MDR-associated patterns, see legend), light red (i.e., FP inhibitors, macrolides, and aminocyclitol with three other MDR-associated patterns) and light blue (i.e., penicillins, cephalosporins, tetracyclines, phenicols, and macrolides with one more MDR-associated pattern). Vertical bars on the far right identify the host origin of *Salmonella enterica* spp. isolates.

The collection of 240 NTS isolates was resolved into 139 PFGE pulsotypes and further grouped into ten clusters (Fig 1). The phylogeny of the NTS demonstrated that the bovine isolates were resolved into three monophyletic clusters with genetic similarity ranging from 75% to 88% within these three clusters. In total, 41 pulsotypes were found among the bovine isolates. Two of these pulsotypes comprised sixteen and eight isolates. Both were found within the second monophyletic cluster. Another pulsotype, comprising six isolates, was identified within the third cluster, whereas the rest of the bovine isolates formed the pulsotypes composed of single, double or triple isolates (Fig 1). The avian isolates were clustered into two paraphyletic clusters with 62% genetic similarity between these two clusters, indicating that the avian isolates were the most heterogeneous group among the examined collection of NTS isolates. The phylogeny demonstrated that the population of avian isolates was resolved into 33 pulsotypes (Fig 1). Three pulsotypes comprising twenty, eight and five isolates were identified in one of the paraphyletic clusters, whereas in another paraphyletic cluster, a pulsotype comprising six isolates was identified (Fig 1). These results provide strong evidence that the population of the avian isolates contains several closely and one distantly related clone. The porcine isolates were also resolved into two paraphyletic clusters with 66% genetic similarity between them (Fig 1). The population of these isolates was resolved into 65 pulsotypes, indicating a non-clonal nature of this group of NTS isolates. Indeed, no clones were identified among the porcine isolates (Fig 1).

### Trends in antimicrobial resistance among the population of NTS isolates obtained from avian, bovine and porcine hosts

**Fig 2:**
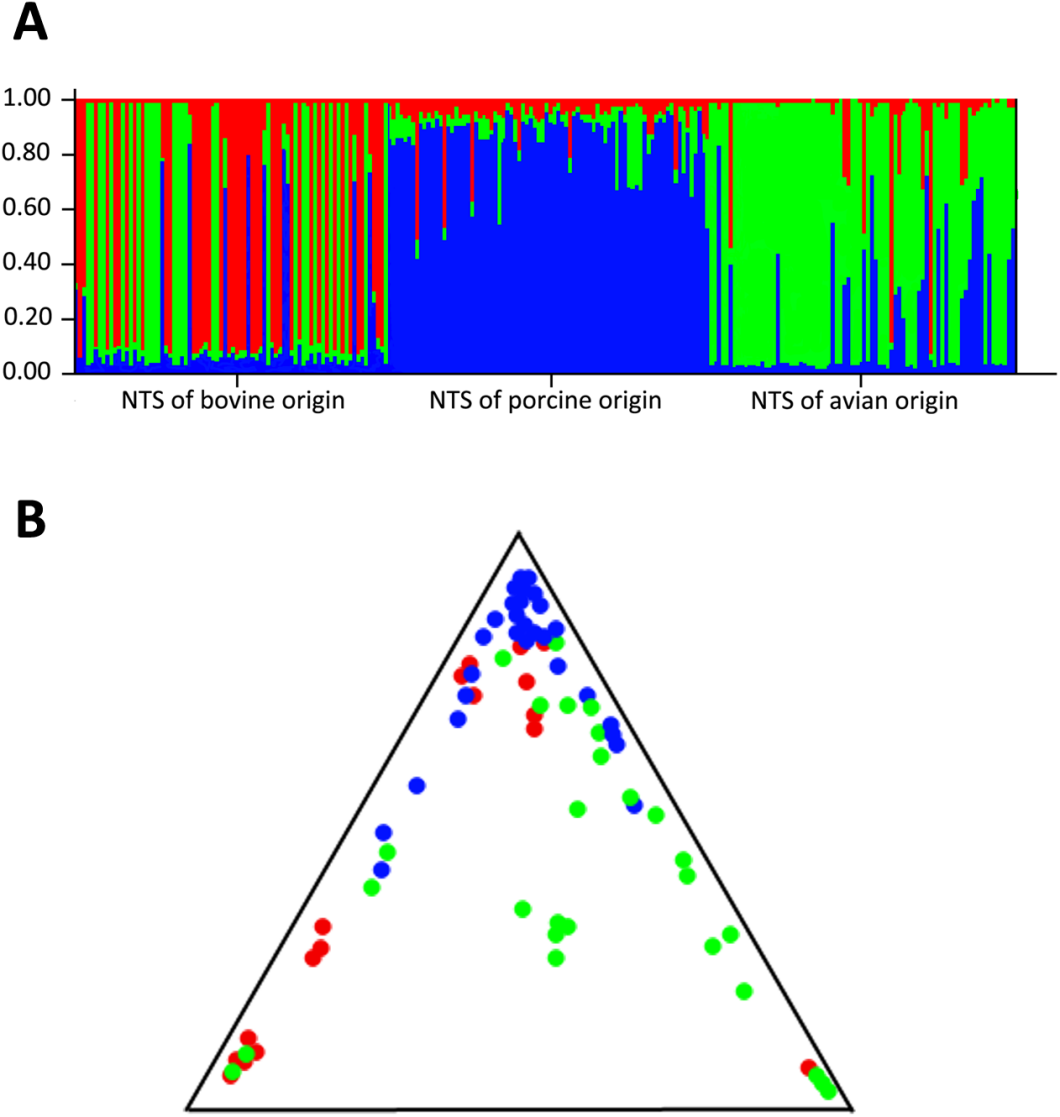
Population structure based on antimicrobial phenotypes of 240 non-typhoidal *Salmonella* isolates obtained from avian, bovine and porcine hosts. **A)** The entire collection of *Salmonella* isolates consisted of three clusters (K 3). The three clusters are color-coded: cluster A is red, cluster B is blue, and cluster C is green. The values along the “y” axis represent the membership probabilities for the three clusters. Each isolate is represented by a single vertical line partitioned into three segments, representing the antimicrobial phenotype admixture that reflects the overall membership in each of these three clusters. **B)** Triangle plot of a Bayesian cluster analysis showing the distribution of avian (green), porcine (blue) and bovine (red) isolates within the examined collection of NTS.

The evolutionary analysis STRUCTURE, based on the antimicrobial profiles of the tested NTS isolates, showed that most isolates were assigned to one of the clusters, creating three asymmetric groups (Fig 2A), which strongly suggests that the collection of NTS isolates is composed of three distinct populations (K = 3). The most significant association between the origin of isolates and the antimicrobial phenotypes was observed for the porcine isolates (85.4%), followed by the avian isolates (73.1%). The isolates of bovine origin showed that 49.0% of the population had a unique antimicrobial phenotype, specific only for the isolates of bovine origin, whereas 36.5% and 14.6% of the antimicrobial phenotypes were associated with the avian and porcine isolates, respectively (Fig 2B). Furthermore, these unique antimicrobial phenotypes created MDR patterns that were specific for each of the host-origin group of NTS isolates. Most notably, each of the host-origin groups of NTS isolates had a distinct MDR pattern with high prevalence. An MDR pattern, involving penicillins, tetracycline and macrolides, was significantly (P < 0.005) associated with the porcine isolates, see Table 2. Two MDR additional patterns, including i) folate pathway inhibitors, macrolide and aminocyclitol, and ii) penicillins, cephalosporin, tetracycline, phenicol and macrolide, showed the highly significant association with the avian (P < 0.005) and bovine (P < 0.005) isolates, respectively, see Table 2. Analyzing the MDR patterns for each host group, we found that these major MDR patterns evolved by acquiring the additional antimicrobial resistance traits. Fifteen MDR patterns, predominantly found in the porcine isolates, were associated with the major MDR pattern found among isolates of porcine origin. The porcine MDR pattern and the fifteen corresponding MDR patterns were significantly (P < 0.005) associated only with the NTS isolates of porcine origin. Similarly, the major avian and bovine MDR patterns, with the corresponding evolved counterparts, were significantly associated with NTS isolates of avian (P < 0.005) and bovine **(P < 0.005**) origin, respectively. Three major MDR patterns (i.e., MDR patterns unique for porcine, avian or bovine isolates), together with the associated MDR patterns, were depicted across NTS pulsotypes and shown in Fig 1.

## Discussion

The emergence and dissemination of antimicrobial resistance poses an enormous threat to humans and food-producing animals worldwide. The common use of veterinary antibiotics, especially the large-scale addition of antibiotics to feed as growth promoters for food-producing animals, has contributed to the emergence and spread of antimicrobial resistant organisms [20–22]. This heightens the need for comprehensive surveillances, designed to determine trends in the emergence and spread of antimicrobial resistance, especially among zoonotic pathogens such as NTS. In the present study, we identified distinct MDR patterns of high frequencies and the evolving counterparts, each strictly associated with NTS isolates of certain host origin, providing compelling evidence that different livestock environments have specific and significant roles in the emergence and spread of MDR organisms. In addition, sequencing the full-length *gyrA* and *gyrB* genes, we were able to identify a novel non-synonymous mutation, located outside of the QRDR at position 864 of the GyrA protein that contributes to fluoroquinolone resistance of NTS isolates.

The most recent trends in the evolution of antimicrobial resistance among the population of *Salmonella enterica* spp. indicate a major replacement of non-multidrug-resistant lineages with epidemic lineages that possess the MDR phenotypes [6, 13, 23] worldwide. Our data show not only the high prevalence rate of MDR phenotypes among NTS isolates obtained from different food-producing animals but also highlight the existence of distinct and frequent MDR phenotypes among each NTS host-associated group. For instance, the MDR phenotype, containing folate pathway (FP) inhibitors, macrolides and aminocyclitol together with the three closely associated (i.e., evolved) counterparts (i.e., [i] FP inhibitors, macrolides, and aminocyclitol + penicillins; [ii] FP inhibitors, macrolides, aminocyclitol + penicillins, and cephalosporins; and [iii] FP inhibitors, macrolides, and aminocyclitol + tetracycline) appear in 72.5% (n = 37) of multidrug-resistant isolates of avian origin, whereas no single isolate with any of these four MDR phenotypes was identified among the isolates of bovine and porcine origin. A similar level of specificity and high prevalence rate exist between the distinct MDR phenotypes and NTS isolates of bovine and porcine origin, which strongly indicates that the host origin (i.e., livestock environment) plays a specific and profound role in the emergence and spread of MDR phenotypes. It is important to mention that the specific MDR phenotypes associated with the host origin of NTS are not acquired by certain *Salmonella* serovars due to unique genetic backgrounds but rather to the existence of specific selective pressures associated with each of the livestock environments. Performing STRUCTURE evolutionary analysis, we found that the NTS isolates of porcine origin, although comprising two serovars (i.e., Typhimurium var 5- [n = 40] and serovar 4,5,12:i:–[n = 40]), showed that the great majority (85.4%) of them had a unique antibiogram, specific only to isolates of porcine origin. Using the same analysis, we found that the avian isolates, although composed entirely of one serovar (i.e., Heidelberg), shared the specific MDR pattern at a lower level (77%) compared to that of the porcine isolates. This evolutionary analysis clearly indicated that the host origin (i.e., livestock environment) was the most determinative factor for acquisition and spread of MDR phenotypes among the tested NTS isolates. Taken together, it can be assumed that different livestock management techniques, particularly the usage of different antibiotics for each food-animal producing group, led to the exposure of NTS isolates to unique selective pressures, which subsequently results in the acquisition and spread of distinct MDR phenotypes among NTS host-associated isolates. Recently, it has been shown that the use of chloramphenicol (Cm) for the treatment of severe bacterial infections in sub-Saharan African regions contributed to a complete replacement of the multidrug-resistant lineage of *S*. Typhimurium Cm-sensitive ST313, with a Cm-resistant ST313 variant [23–25], providing strong evidence of the detrimental effect of selective pressure on the evolution of antimicrobial resistance in a population of NTS strains.

The phylogeny of the tested NTS isolates in our study demonstrated that these host-specific and highly frequent MDR phenotypes have been vertically transmitted to descendants, further suggesting the presence of constant and unique selective pressures within the hosts of NTS isolates over a long time period. For instance, the phylogeny and antibiogram of the entire monophyletic cluster of serovar Typhimurium var 5 (Fig 1; marked by an asterisk), demonstrated that each of the 21 pulsotypes of this cluster possessed a unique porcine MDR phenotype (i.e., evolved penicillins, tetracyclines, and macrolides MDR pattern), suggesting vertical transmission of these MDR phenotypes (Fig 1). This is another line of evidence indicating that the livestock environments have a specific and significant effect on the evolution of MDR phenotypes among NTS strains associated with these environments. Interestingly, the population of *Salmonella* serovar Typhimurium var 5 isolates possessed only the evolved porcine MDR patterns (i.e., penicillins, tetracyclines, and macrolides + phenicols; penicillins, tetracyclines, macrolides + phenicols, aminocyclitol, etc.), whereas most (67%) of the MDR *Salmonella* serovar 4,5,12:i:–isolates contained the basic porcine MDR phenotype (i.e., penicillins, tetracyclines, and macrolides). This phenomenon can be explained by a temporal effect of the selective pressure on these two *Salmonella* populations, serovars 4,5,12:i:–and Typhimurium var 5. In other words, both *Salmonella* serovars were exposed to the same selective pressure that resulted in the acquisition of the MDR phenotypes specific for the porcine isolates, but their exposures were most likely over a different period of time. The population of serovar Typhimurium var 5 isolates was most likely exposed to this selective pressure over a longer period of time compared to the case of serovar 4,5,12:i:–, which resulted in the basic MDR pattern (i.e., penicillins, tetracyclines, and macrolides) that evolved through the acquisition of additional antibiotic resistant genes. Most recently, Feasey et al. (6) reported that in sub-Saharan Africa regions, the smallest of the known *Salmonella* virulence-associated plasmids, pSENV, nearly doubled in size, partly through the acquisition of antibiotic resistant genes. A similar process of evolution of MDR phenotypes most likely occurred among the NTS isolates associated with the livestock environments.

Although fluoroquinolones (FQs) are recommended as first-line drugs for the treatment of iNTS infections, several studies imply that the incidence of FQ-resistant NTS is increasing worldwide [26–28]. As most FQs resistance-associated mutations occur in a region of the *gyrA* gene known as the quinolone resistance-determining region (QRDR) (i.e., for *Salmonella enterica* spp. amino acids 67–112), numerous studies reported FQ resistance-associated mutations within the QRDR [29–33]. In this study, by sequencing the entire length of the *gyrA* and *gyrB* genes of a large collection of NTS isolates, we identified a unique non-synonymous mutation in *gyrA*, which is located outside of the QRDR. This novel, likely FQ resistance-associated mutation, occurred close to the C terminus of the GyrA protein, at position 864, where serine was substituted proline, indicating the importance of this GyrA region in the evolution of FQ resistance in the population of NTS isolates.

In conclusion, the present study, which tested a large, well-defined host origin population of NTS isolates, clearly demonstrated that the livestock environment has a specific and profound role in the evolution of MDR phenotypes in this zoonotic pathogen. These findings imply that each livestock environment (i.e., avian, bovine and porcine) has a unique set of selective pressures that cause the acquisition and spread of distinct antimicrobial resistant genes in NTS associated with that environment. Our data showed that the major MDR phenotypes reflect the particular livestock environment. The findings from this study should be considered in intensively managed livestock operations. In addition to providing current insight into the trends in antibiotic susceptibility of NTS isolates, this study described a novel non-synonymous mutation, located outside of the QRDR at position 864 of GyrA, that is likely associated with FQ resistance.

## Material and methods

### Collection of non-typhoidal *Salmonella enterica*

In total, 240 NTS isolates were collected, including 80 clinical isolates of bovine origin (i.e., *S*. *enterica* serovar Dublin [n = 50]; *S*. *enterica* serovar Cerro [n = 16]; and *S*. *enterica* serovar Montevideo [n = 14]), 80 clinical isolates of porcine origin (i.e., *S*. *enterica* serovar Typhimurium var 5- [n = 40] and *S*. *enterica* serovar 4,5,12:i:–[n = 40]) and 80 isolates associated with poultry barns (i.e., *S*. *enterica* serovar Heidelberg [n = 80]). The porcine isolates were collected during 2015 from fourteen US states, including: Arkansas, Colorado, Illinois, Iowa, Kansas, Kentucky, Minnesota, Michigan, Missouri, Nebraska, Ohio, Oklahoma, South Carolina and Texas. Each isolate was obtained from a single animal (i.e., no multiple isolates per an animal) and each animal was housed in a distinct swine farm (i.e., no two animals from the same farm). The bovine isolates were also collected during 2015 from five states, including: Indiana, Iowa, Kansas, Minnesota and Wisconsin. Similarly, each bovine isolate was obtained from a single animal that were housed in 70 farms scattered across these five states. The avian isolates were collected during 2015–2106 from Minnesota. Each avian isolate was obtained from a distinct sample/farm. Isolates of bovine and porcine origin were received from the Veterinary Diagnostic Laboratory (VDL), and isolates of the avian origin were received from the Minnesota Poultry Testing Laboratory (MPTL). Primary identification occurred at the National Reference Laboratory, Ames, Iowa, using standard microbiological and serological methods. Isolates were stored at –80°C in Luria-Bertani (LB) broth (Difco) containing 10% glycerol. For each experiment in this study, fresh cultures derived from the frozen stocks were used.

### Antimicrobial susceptibility testing

The entire collection of NTS isolates was examined for antibiotic resistance determinants using Sensititre BOPO6F plates (TREK Diagnostic Systems, Cleveland, OH). The Sensititre plates each contained 18 antimicrobial agents: ceftiofur (8–0.25 μg/mL), tiamulin (32–1 μg/mL), chlortetracycline (8–0.5 μg/mL), florfenicol (8–0.25 μg/mL), oxytetracycline (8–0.5 μg/mL), penicillin (8–0.12 μg/mL), ampicillin (16–0.25 μg/mL), danofloxacin (1–0.12 μg/mL), sulfadimethoxine (256 μg/mL), neomycin (32–4 μg/mL), trimethoprim/sulfamethoxazole (2/38 μg/mL), spectinomycin (64–8 μg/mL) and enrofloxacin (2–0.12 μg/mL), dosed in 96 wells at the appropriate dilutions specified by the NARMS (National Antimicrobial Resistance Monitoring System) of the CDC. Each well of the Sensititre plate was inoculated and incubated according to the manufacturer’s instructions. The minimal inhibitory concentration (MIC) was manually determined for each NTS strain as the lowest concentration of each antibiotic that inhibited visible growth. MIC breakpoints were determined according to the National Committee for Clinical Laboratory Standards (NCCLS) m100 [34] and M31 [35].

### DNA extraction

Non-typhoidal *Salmonella* isolates were plated from frozen stocks on Luria-Bertani (LB) medium (Difco), followed by overnight incubation at 37°C. Growth from an overnight culture was collected with a sterile loop and resuspended in 1 mL of 0.9% saline. After centrifugation at 10000 X g for 1 min, the supernatant was removed, and genomic DNA was extracted using the Qiagen DNeasy tissue kit (Qiagen Inc., Valencia, CA) according to the manufacturer’s instructions.

### Sequencing of full-length *gyrA* and *gyrB* genes

Primers for the *gyrA* and *gyrB* genes, *gyrA* (forward 5’–GGC TGC ATT CCG TTT ACC A and reverse 5’–GGA TAT CCG GCC CTC GCA CAG C) and *gyrB* (forward 5’–AAA AGG GTA AAA TAA CGG ATT and reverse 5’–CGA TGA ACA TCA TGA TGC CC), were designed to flank ~ 90 bp up- and down-stream of the targeted genes. The amplicons were generated by Platinum *Taq* DNA polymerase (Thermo Fisher Scientific) and were prepared for DNA sequencing by the Prism BigDye Terminator cycle sequencing kit (Applied Biosystems). Amplicon sequencing was carried out with the following primers: *gyrA*-F1 (-87 bp; 5’–GGC TGC ATT CCG TTT ACC A), *gyrA*-F2 (445 bp; 5’–CTA TGA CGG TAC GGA AAA AAT), *gyrA*-F3 (971 bp; 5’–GAC CCA GCT ACA GGT TTC CTT C), *gyrA*-F4 (1507 bp; 5’—ATC CGC GAA GAG ATG GAG TTA), *gyrA*-F5 (2023 bp; 5’–GGT GAA CCT CAA CGA CGG C), *gyrB*-F1 (-51 bp; 5’—AAA AGG GTA AAA TAA CGG ATT), *gyrB*-F2 (531 bp; 5’–CCA ACG TCA CTG AAT TTG AAT ATG A), *gyrB*-F3 (1062 bp; 5’–AAC GAA CTG CTG AGC GAA TAC C), and *gyrb*-F4 (1667 bp; 5’- ACC AGA TTT CCA TCG CGC TTG ACG G). The nucleotide sequences were determined using an ABI 3730x1 DNA analyzer (Genomic Center, University of Minnesota, Minneapolis, MN). Each strand containing a SNP was aligned with the complementary strand. A consensus DNA sequence was obtained using Clustal Omega [36]. The annotated DNA sequences were exported into Molecular Evolutionary Genetics Analysis (MEGA) version 7 [37] for the identification of nsSNPs. Nucleotide sequence translation was carried out using EMBOSS Transeq [38] (European Molecular Biology Laboratory–European Bioinformatics Institute; Hinxton, Cambridge, United Kingdom).

### Molecular typing

All NTS isolates were characterized by the PFGE typing method, as previously described by the Centers for Disease Control and Prevention (CDC) PulseNet program [39, 40]. Briefly, genomic DNA was digested with the restriction enzyme XbaI (New England Biolabs Inc., Beverly MA) overnight at 25°C. Electrophoresis was carried out using a 1% agarose gel in 0.5 X Tris-borate-EDTA buffer at 14°C with the following conditions: 6 V/cm for 19 h, with an initial switch time of 2.16 s and final switch time of 63.8 s. The PFGE patterns were analyzed using BioNumerics software version 5 (Applied Maths, St-Martens-Latern, Belgium). Similarities between PFGE patterns were determined based on the Dice similarity coefficient. The resulting similarities in the matrix were further processed by employing the unweighted-pair group method using average linkages to create a dendrogram that depicted the genetic relatedness between NTS isolates.

### Population structure analysis

The program STRUCTURE [41] was used to investigate the population structure of NTS isolates based on associations with eight antibiotic categories including: penicillins, cephalosporins, tetracyclines, fluoroquinolones, phenicols, folate pathway inhibitors, macrolides and aminocyclitols. A Bayesian model approach was used to infer population structure (K) within the studied collection of NTS isolates and to individually assign isolates to the best-fitting population. K values were evaluated by a Markov chain Monte Carlo algorithm with a 10 model-run. Each of the 10 runs consisted of a 10,000 burn-in period, followed by 100,000 reps using the admixture model. The programs CLUMPP [42] and DISTRUCT [43] were used to generate the initial graphs depicting the antimicrobial resistance phenotype distribution across the NTS isolates of avian, bovine and porcine origin.

### Statistical analysis

For each antibiotic, association between resistance status (R vs not R) and host type was tested using a chi-squared test. For each multidrug resistant pattern separately, association with host type was tested using a chi-squared test, with the null hypothesis being that the multidrug resistant pattern was equally likely to occur in each host type, and p-values were corrected for multiple comparison with the Bonferroni-Holm adjustment. Only multidrug resistant patterns with three or more occurrences were included.

### Nucleotide sequence accession numbers

Nucleotide sequences were deposited in GenBank. Accession numbers for the DNA sequences of the *gyrA* and *gyrB* genes ranged from KY611563 to KY611579.

## Supporting information

S1 File (TXT)

S2 File (TXT)

S3 File (TXT)

S4 File (TXT)

S5 File (TXT)

S6 File (TXT)
